# Copper Uptake in Mammary Epithelial Cells Activates Cyclins and Triggers Antioxidant Response

**DOI:** 10.1155/2015/162876

**Published:** 2015-10-25

**Authors:** Nathália Villa dos Santos, Andreza Cândido Matias, Guilherme Shigueto Vilar Higa, Alexandre Hiroaki Kihara, Giselle Cerchiaro

**Affiliations:** ^1^Center for Natural Sciences and Humanities, Federal University of ABC (UFABC), 09210-170 Santo André, SP, Brazil; ^2^Center for Mathematics, Computation and Cognition, Federal University of ABC (UFABC), 09210-170 Santo André, SP, Brazil

## Abstract

The toxicologic effects of copper (Cu) on tumor cells have been studied during the past decades, and it is suggested that Cu ion may trigger antiproliferative effects *in vitro*. However, in normal cells the toxicologic effects of high exposures of free Cu are not well understood. In this work, Cu uptake, the expression of genes associated with cell cycle regulation, and the levels of ROS production and related oxidative processes were evaluated in Cu-treated mammary epithelial MCF10A nontumoral cells. We have shown that the Cu additive is associated with the activation of cyclin D1 and cyclin B1, as well as cyclin-dependent kinase 2 (CDK2). These nontumor cells respond to Cu-induced changes in the oxidative balance by increase of the levels of reduced intracellular glutathione (GSH), decrease of reactive oxygen species (ROS) generation, and accumulation during progression of the cell cycle, thus preventing the cell abnormal proliferation or death. Taken together, our findings revealed an effect that contributes to prevent a possible damage of normal cells exposed to chemotherapeutic effects of drugs containing the Cu ion.

## 1. Introduction

Recent advances in biochemical tools have highlighted the extraordinary array of functions of Cu in living organisms [1]. Cu is required for binding to the dual specificity mitogen-activated protein kinase kinase 1* MEK1* and promotion of mitogen-activated protein kinase* MAPK* signaling and tumorigenesis by v-raf murine sarcoma viral oncogene homolog B* BRAF* in mammary tumors [2]. However, despite the enormous expansion in our knowledge of Cu biology that has occurred over the last decades, we are only just beginning to unravel the complexity of the role of this transition metal in the regulation of cellular processes and cell cycle.

Cu is an essential trace element and its intracellular concentrations are tightly controlled. Within the cell, Cu is distributed by metallochaperones and plays a fundamental role in regulating cell growth, altering gene expression (due to oxidation of guanosine and adenosine residues in nucleic acids or changes in transcription factor/growth factor activities) [3]. A critical factor in the development of cancer is angiogenesis, which endows continuous supplying of nutrients, growth factors, and signaling agents to malignant tissue [4–7]. This angiogenic response in tumor is stimulated by ceruloplasmin, the plasma Cu-carrier [6, 8, 9]. Although these studies with cancer cells and tumors strongly suggested that Cu plays an essential role in cell growth and proliferation, little is known about underlying molecular mechanisms. Also, Cu is involved in redox reactions that generate intracellular reactive oxygen species (ROS), mainly by Fenton reaction, and a number of reports point to a relationship between Cu, ROS production, and cancer development [10, 11], and recently the role of Cu metabolism in resistance of cancer cells to cisplatin [12–14]. The redox status of cells is influenced by the homeostasis of reactive species, since ROS might act as secondary messengers in the regulation of pathways associated with cell proliferation, differentiation, and apoptosis [15, 16]. Based on these findings, some studies suggested that elevated Cu levels and increased oxidative stress may be used in selective cancer therapy [17, 18]; however, the effect of Cu-stimulation in cell proliferation and its relationship with ROS needs to be well elucidated, especially in nontumoral cells.

The aim of the present study was to clarify the connection of the Cu with cell cycle activation in normal epithelial cells and to determine the mechanism by which this ion, supplied as CuSO_4_, stimulates the cell cycle of breast epithelial cells* in vitro*. For this purpose, the Cu uptake, the expression of genes associated with cell cycle regulation, and the levels of ROS production and related oxidative processes were evaluated in Cu-treated mammary epithelial MCF10A nontumoral cells.

## 2. Methods

### 2.1. Chemicals

Unless otherwise stated, chemicals were obtained from Sigma-Aldrich (USA) and were of analytical grade: solutions were prepared using Milli-Q water (Millipore, Billerica, USA).

### 2.2. Cell Cultures

Cell media were prepared with DNAse- and RNAse-free water and filtered through 0.22 *μ*m filter membranes (Millex GV, SLGV033RS, Millipore, Billerica, USA) prior to use. Cell cultures were manipulated using sterile, disposable nonpyrogenic plastic ware and were maintained at 37°C in an atmosphere of 5% CO_2_ in air at a relative humidity of 80%. Human breast epithelial cells MCF-10A (ATCC) were cultured in a 1 : 1 (v/v) mixture of Dulbecco's Modified Eagle's Medium (DMEM, 12100046, Gibco, Waltham, USA) and Ham's F12 nutrient mixture (HamF12, 21700-075, Gibco, Waltham, USA) supplemented with 5% inactivated horse serum (16050-130, Gibco, Waltham, USA) 10 *μ*g/mL insulin (PHC9624, Gibco, Waltham, USA), 0.020 *μ*g/mL human epidermal growth factor (EGF, PHG0311, Gibco, Waltham, USA), 0.5 *μ*g/mL hydrocortisone (H0888, Sigma Aldrich, St. Louis, USA), 0.10 *μ*g/mL choleric toxin (C8052, Sigma Aldrich, St. Louis, USA), 100 U/mL penicillin, and 100 *μ*g/mL streptomycin (P4333, Sigma Aldrich, St. Louis, USA). Cells were routinely trypsinized and inoculated onto plates at a density of 4 × 10^4^ cells/cm^2^. Every month, cells were cultivated in the absence of antibiotics for control purposes and subjected to routine assay using a MycoAlert Mycoplasma Detection kit (LT07, Lonza, Walkersville, USA).

### 2.3. Cell Proliferation Assays

MCF-10A cells were incubated in 24-well plates at a density of 4 × 10^4^ cell/cm^2^ for the period of 24 h under the conditions described above. Aliquots of freshly prepared solutions of CuSO_4_ were added separately to the culture medium (less than 1% of total volume) in order to attain final concentrations in the range 25.0–1000 *μ*M, and the plates were incubated for further 24–48 h. On the basis of the results obtained subsequent experiments were conducted by incubating treated cells to CuSO_4_ at final concentrations of 50 *μ*M (*n* = 5) and control cells on unsupplemented medium. Typically, cells were plated onto the medium at a density of 4 × 10^4^ cells/cm^2^ to give monolayers of approximately 50–60% cell confluence and incubated for 48 h. Following incubation, cells were trypsinized, washed with phosphate buffered saline (PBS: 137 mM NaCl and 2.7 mM KCl in 10 mM phosphate buffer at pH 7.4), stained with Trypan Blue (T8154, Sigma Aldrich, St. Louis, USA), and counted under an optical microscope using a Neubauer's chamber [19].

### 2.4. Isolation of RNA, Synthesis of cDNA, and Real-Time PCR

MCF10A cells that had been plated and incubated in the presence or absence of CuSO_4_ (50 *μ*M, *n* = 6) were homogenized in 1 mL TRIzol reagent (15596-026, Invitrogen, Waltham, USA) and total RNA extracted according to the protocols of the manufacturer. After air-drying, RNA was resuspended in diethylpyrocarbonate-treated water (DEPC, 40718, Sigma Aldrich, St. Louis, USA) and the concentration determined from the absorbance at 260 nm. Residual DNA was removed using DNase I (E2215Y, GE Healthcare Life Sciences, USA) following the protocol of the manufacturer. For each reverse transcription reaction, 4 *μ*g of total RNA was mixed with 1 *μ*L oligo(dT) [11–17] primer (0.5 *μ*g/*μ*L; Invitrogen, Waltham, USA) and incubated for 10 minutes at 65°C. The mixture was then chilled on ice, mixed with 4 *μ*L of 5x first strand buffer, 2 *μ*L of 0.1 M DTT (R0861, Thermo Scientific, Waltham, USA) 1 *μ*L of dATP, dTTP, dCTP, and dGTP (each 10 Mm, AM8200, Invitrogen, Waltham, USA), 1 *μ*L of SuperScript III reverse transcriptase (200 U; 18080-044, Invitrogen, Waltham, USA), and sterile water to a final volume of 20 *μ*L, incubated for 60 minutes at 50°C and subsequently inactivated by heating at 70°C for 15 minutes. Real-time PCR was carried out using a Corbett Life Science Rotor-Gene 6000 thermal cycler (Qiagen) with specific primers for human cyclin D1 (forward: 5′-TGGGTCTGT GCATTTCTGGTT-3′, reverse: 5′-GCTGGAA ACATGCCGGTTAC-3′) and cyclin B1 (forward: 5′-AGGAACTCGAAAATTAATGCT GAAA-3′, reverse: 5′-CCGTAGGAACGCGC TTTG-3′). As an internal control, glyceraldehyde 3-phosphate dehydrogenase (GAPDH) gene expression was determined with specific primers (forward: 5′-CCACCCATGGC AAATTCC-3′, reverse: 5′-TGGGATTTCCATT GATGACAAG-3′). PCR assays were performed using the following program: carryover prevention for 2 minutes at 50°C and initial activation for 10 minutes at 95°C, followed by two-step cycling for 10 seconds at 95°C (denaturation) and 1 minute at 60°C (annealing/extension). Dissociation curves of PCR products were obtained by heating samples from 60 to 95°C in order to evaluate primer specificity.

### 2.5. Analysis of PCR Data

The relative levels of expression of target genes were evaluated using the comparative cycle threshold method as described by Medhurst et al. [20]. A value for *C*_*T*_ was determined from the fluorescence detected within the geometric region of the semilog amplification plot and represented the PCR cycle number at which fluorescence was detectable above an arbitrary threshold established on the basis of the variability of baseline data during the first 15 cycles.

### 2.6. Solid Sampling in Graphite Furnace Atomic Absorption Spectroscopy (GFAAS)

Experimental parameters were obtained from Carvalho Do Lago et al. [21] and the new developed methodology with dried cells [20]. Briefly, a model ZEEnit 600 (Analytik Jena) atomic absorption spectrometer, equipped with a transversely heated graphite atomizer, an inverse and transversal 2- and 3-field mode Zeeman effect background corrector, manual sampling accessory, and hollow Cu cathode lamp, was employed to determine intracellular Cu concentrations. Pyrolytically coated heated graphite tubes and pyrolytically coated boat-type solid sampling platforms were employed throughout. Argon (99.998% v/v; Air Liquide, Mauá, Brasil) was used as protective and purge gas. All measurements were based on integrated absorbance values acquired with the aid of Windows NT software.

### 2.7. Determination of Cu Content of Cultured Cells

MCF10A cells that had been plated and incubated in the presence or absence of CuSO_4_ (50.0 *μ*M) as described above were trypsinized and adherent cells were combined, washed five times with phosphate buffered saline (PBS: 137 mM NaCl and 2.7 mM KCl in 10 mM phosphate buffer at pH 7.4) containing 1.0 mM EDTA in order to remove residual Cu(II), and dried for 1 week in a desiccator. Experiments were conducted in triplicate or quintuplicate using plates of surface area 75 or 150 cm^2^. For the GFAAS determination of Cu, procedures were followed as from Carvalho Do Lago et al. [21].

### 2.8. Extraction of Nuclei

Nuclei from MCF10A cells that had been incubated in the presence or absence of CuSO_4_ (50.0 *μ*M) as described above were isolated using published procedures [22, 23]. Briefly, cells were inoculated at a density of 4 × 10^4^ cell/cm^2^ into culture bottles containing appropriate medium (150 cm^2^ surface area of culture) and incubated for 48 hours at 37°C in an atmosphere of 5% CO_2_ in air at a relative humidity of 80%. Experiments were conducted in quadruplicate. Following incubation, cells were trypsinized and adherent cells were combined, washed with PBS, and centrifuged (250–300 ×g, 10 minutes, 4°C) and the pellet was resuspended in 2 mL of lysis buffer (10 mM NaCl, 3 mM MgCl_2_, and 0.5% Tergitol NP-40 in 10 mM Tris buffer at pH 7.5) and left on ice for 5 minutes. Cells were subsequently centrifuged (500 ×g, 5 minutes, 4°C) and the pellet was resuspended in 2 mL of lysis buffer and recentrifuged. The pellet from the second centrifugation (containing extracted nuclei) was dried in an oven at 30°C and subsequently analyzed by GFAAS. The purities of the extracted nuclei were determined by Western blot analyses using rabbit antihistone H3 N-terminal and rabbit anti-human Cu/Zn SOD1 polyclonal antibodies.

### 2.9. Generation of Intracellular Reactive Oxygen Species

MCF10A cells that had been plated and incubated in the presence or absence of CuSO_4_ (50.0 *μ*M) were treated with trypsin/EDTA (1 mM, 25200-056, Gibco, Waltham, USA) solution, washed three times with PBS, and suspended in a 50.0 *μ*M solution of the* oxidation-sensitive* nonfluorescent probe 2′,7′-dichlorodihydrofluorescein diacetate (DCFH, D6883, Sigma Aldrich, St. Louis, USA) [24, 25]. Following incubation at 37°C for 45 minutes [19], the cells were washed three times with PBS and the levels of intracellular fluorescence were determined immediately by flow cytometry at 530 nm using a Cytomics FC 500 MPL (Beckman Coulter) instrument [26, 27]. Assays were conducted at least in quintuplicate and >20,000 viable cells from each sample were analyzed per assay, in arbitrary units of fluorescence.

### 2.10. Determination of GSH/GSSG Ratio

MCF10A cells that had been plated and incubated in the presence or absence of CuSO_4_ (50.0 *μ*M) as described above were trypsinized and adherent cells were combined, washed three times with cold PBS, and centrifuged (1500 ×g, 3 minutes, 4°C). The cells were resuspended in 400 *μ*L of cold water and lysed by freezing in a mixture of dry ice and ethanol for 10 minutes. After thawing, proteins were precipitated with 100 *μ*L of 10% sulfosalicylic acid (390275, Sigma Aldrich, St. Louis, USA) and centrifuged (4000 ×g, 5 minutes, 4°C). The protein concentration in the pellet was determined, and the levels of GSH and GSSG in the supernatant were assessed using the protocol of Martín et al. [28]. For GSH quantification, the assay mixture contained 100 *μ*L of supernatant, 790 *μ*L of a 0.1 M sodium phosphate buffer containing 0.05% EDTA at pH 7.0, 100 *μ*L of 6 mM 5,5′-dithiobis (2-nitrobenzoic acid) (DTNB, D8130, Sigma Aldrich, St. Louis, USA) dissolved in glutathione assay buffer (GAB; 125 mM sodium phosphate containing 6.3 mM EDTA), and 10 *μ*L glutathione reductase (55 U/mL, G3664, Sigma Aldrich, St. Louis, USA). For GSSG quantification, the assay mixture comprised 100 *μ*L of supernatant, 190 *μ*L of 0.5 M phosphate buffer at pH 6.8, 700 *μ*L of 0.3 mM NADPH (N5130, Sigma Aldrich, St. Louis, USA), prepared in GAB, and 10 *μ*L of glutathione reductase (55 U/mL). The reaction rate was estimated from the change in absorbance at 412 nm after 3 minutes at 25°C (for GSH) or at 340 nm after 16 minutes at 30°C (for GSSG) [28–30]. The accuracy of the GSH reference standard was measured with DTNB (D8130, Sigma Aldrich, St. Louis, USA), using a molar extinction coefficient of 13,600 with an absorbance of 412 nm [31]. GSSG was standardized by measuring the decline of NADPH in the presence of glutathione reductase, taking into consideration that the molar extinction coefficient of NADPH to be 6270 at 340 nm and 1 mol of NADPH converts 1 mol of GSSG to 2 mol of GSH [32].

### 2.11. Western Blot Analyses

MCF10A cells that had been plated and incubated in the presence or absence of CuSO_4_ (50.0 *μ*M) as described above were treated with trypsin/EDTA (1 mM, 25200-056, Gibco, Waltham, USA) solution, washed three times with PBS, resuspended in 150 *μ*L RIPA buffer (150 mM NaCl, 5 mM EDTA, 1 mM dithiothreitol, 1% Triton X-100 (X100, Sigma Aldrich, St. Louis, USA), 0.5% sodium deoxycholate (30970, Sigma Aldrich, St. Louis, USA), and 0.1% SDS (L3771, Sigma Aldrich, St. Louis, USA) in 50 mM Tris at pH 7.5) containing protease inhibitor cocktail for mammalian cells (P8340, Sigma Aldrich, St. Louis, USA), and centrifuged (14000 ×g, 20 min). Supernatants and pellets were transferred to Eppendorf tubes and stored at −80°C until being required for analysis. Protein concentrations were determined according to the method of Lowry [33] using bovine serum albumin (A2153, Sigma Aldrich, St. Louis, USA) as standard, and extracts were submitted to SDS-PAGE and blotted onto nitrocellulose membranes (10600001, GE Healthcare Life Sciences, New York, USA) with equal loading of protein being confirmed by internal mass control blotting of *α*-tubulin (T9026, Sigma Aldrich, St. Louis, USA). Membranes were blocked for 1 hr in blocking solution comprising 5% nonfat-dried milk (M7409, Sigma Aldrich, St. Louis, USA) and 0.0025% sodium azide (V000494, Sigma Aldrich, St. Louis, USA) solubilized in TBS-T (150 mM NaCl, 50 mM Tris at pH 7.5, and 0.05% Tween-20) and washed twice with TBS-T. The primary antibodies employed were mouse anticyclin dependent kinase 2 (anti-CDK2 clone p34 P9OH-1 sc-51578; Santa Cruz Biotechnology, Heidelberg, Germany) and mouse anti-*α* tubulin (DM1A; Santa Cruz Biotechnology, Heidelberg, Germany) monoclonal antibodies or rabbit antihistone H3 N-terminal (H0164, Sigma Aldrich, St. Louis, USA) and rabbit anti-human Cu/Zn superoxide dismutase 1 (AB5480, Millipore, Billerica, USA) polyclonal antibodies. The specific protein complexes formed following treatment with specific secondary antibody (anti-mouse or anti-rabbit IgG-peroxidase conjugate, A4416 or A0545, Sigma Aldrich, St. Louis, USA) were detected using SuperSignal West Pico chemiluminescent substrate (34080, Thermo Scientific, Waltham, USA).

### 2.12. Statistical Analyses

All experiments were repeated at least five times (except where stated otherwise) and the results are expressed as mean values ± standard deviations. Analysis of variance (ANOVA) with the Bonferroni correction was used to evaluate the differences between means with the level of significance set at *p* < 0.05. For real-time PCR experiments, values obtained for each cell lineage were entered into a two-way ANOVA with factors time and treatment, and pairwise comparisons were performed using the Tukey HSD test with the level of significance set at *p* < 0.05.

## 3. Results

### 3.1. Viability *e* Test and Measurement of Intracellular Copper Levels

**Figure 1:**
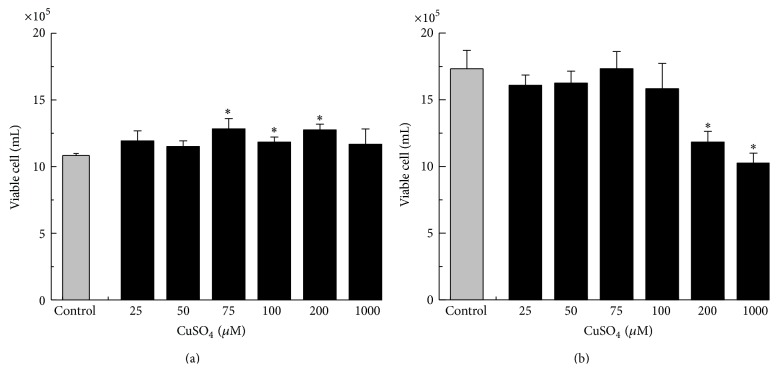
Cu treatment and the proliferation of epithelial MCF10A cells. MCF10A cells were treated with CuSO_4_ for (a) 24 hours and (b) 48 hours, while untreated cells were used as control. Viable cells were counted after staining with Trypan Blue. Data represent mean values ± standard deviation (*n* = 5). Significant differences between Cu-treated and untreated cells are indicated by asterisks (^*∗*^ *p* < 0.05).

The effects of Cu on the viabilities of the nontumor line MCF10A were initially evaluated using the Trypan Blue exclusion test. On the basis of concentration-dependent studies (Figure 1), proliferation of MCF10A was not observed at concentrations above 75.0 *μ*M CuSO_4_ after 24 hr of incubation (Figure 1(a)), but cell viability was significantly reduced after 48 hr (Figure 1(b)) when levels of Cu were equal to or greater than 200.0 *μ*M. We did not observe differences in cell proliferation or cell death when comparing untreated and treated cells after 24 hours, 10.83 ± 0.14 × 10^5^ viable cells/mL* versus *11.91 ± 0.76 (*p* = 7.32 × 10^−2^) for 25 *μ*M Cu, or* versus *11.50 ± 0.43 (*p* = 6.46 × 10^−2^) for 50 *μ*M Cu, or* versus *12.83 ± 0.76 (*p* = 61.12 × 10^−2^) for 75 *μ*M Cu, or* versus *11.83 ± 0.38 (*p* = 1.32 × 10^−2^) for 100 *μ*M Cu, or* versus *12.75 ± 0.43 (*p* = 1.19 × 10^−3^) for 200 *μ*M Cu, or* versus *11.67 ± 1.15 (*p* = 2.82 × 10^−1^) for 1000 *μ*M Cu (Figure 1(a)). Similarly for 48 hours of Cu incubation we did not observe differences in cell proliferation or cell death when comparing untreated and treated cells, 17.33 ± 1.37 × 10^5^ viable cells/mL* versus *16.08 ± 0.76 (*p* = 2.41 × 10^−1^) for 25 *μ*M Cu, or* versus *16.25 ± 0.91 (*p* = 3.17 × 10^−1^) for 50 *μ*M Cu, or* versus *17.33 ± 1.28 (*p* = 1) for 75 *μ*M Cu, or* versus *15.83 ± 1.89 (*p* = 3.29 × 10^−1^) for 100 *μ*M Cu, or* versus *11.83 ± 0.80 (*p* = 3.94 × 10^−3^) for 200 *μ*M Cu, or* versus *10.25 ± 0.75 (*p* = 1.44 × 10^−3^) for 1000 *μ*M Cu (Figure 1(b)). Due to the nonproliferative effect on MCF10A, the concentration of 50.0 *μ*M CuSO_4_ was chosen in whole study.

**Figure 2:**
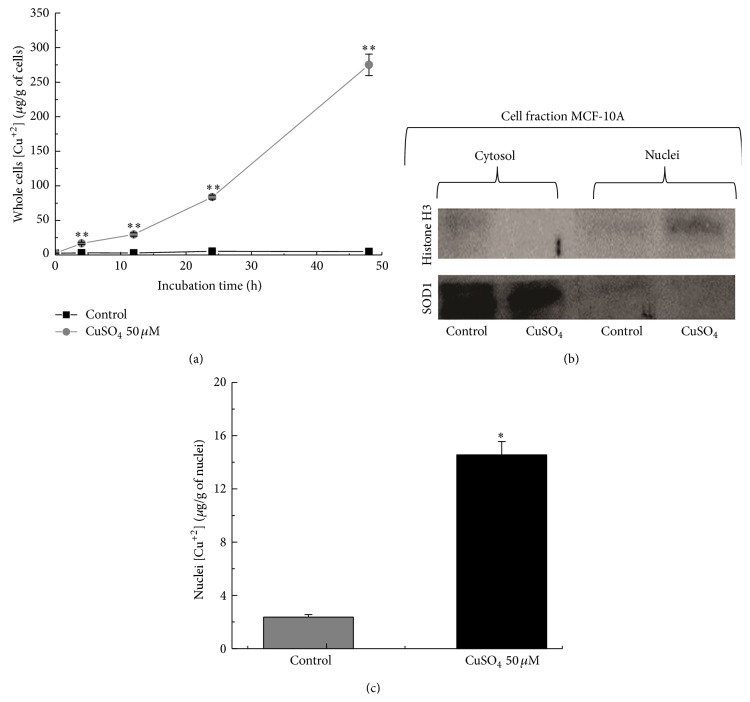
The uptake of Cu during the time, as determined by solid sampling-graphite furnace atomic absorption spectroscopy. (a) Concentration of Cu in whole cells of MCF10A treated with 50 *μ*M CuSO_4_ and in untreated cells, during a period of 48 hours. When compared to controls, cells incubated with 50 *μ*M of CuSO_4_ showed higher Cu(II) levels after 4, 24, and 48 hours. (b) The purities of the nuclei extracted from nontumor MCF10A cells were verified by Western blot analyses. Rabbit antihistone H3 N-terminal and rabbit anti-human Cu/Zn-superoxide dismutase 1 polyclonal antibodies were used to detect the corresponding proteins in the cytosolic and nuclear fractions. (c) Concentration of Cu in the nuclei of controls and cells treated with 50 *μ*M CuSO_4_ after 48 hours. Significant differences between Cu-treated and untreated cells are indicated by asterisks (^*∗*^ *p* < 0.01, ^*∗∗*^ *p* < 0.001).

The Cu uptake from medium supplemented with CuSO_4_ was evaluated by subjecting whole dried MCF10A cells to GFAA [34]. Interestingly, MCF10A cells were able to internalize Cu(II). When compared to controls, cells incubated with 50 *μ*M of CuSO_4_ showed higher Cu(II) levels after 4 hours, 3.07 ± 0.10 *μ*g/g* versus *16.76 ± 1.63 *μ*g/g (*p* = 1.51 × 10^3^), respectively (Figure 2(a)). After 12 hours, differences between untreated and treated cells increased, considering the respective values 2.91 ± 0.14 *μ*g/g* versus *29.61 ± 2.46 *μ*g/g (*p* = 1.99 × 10^−4^, Figure 2(a)). We also observed differences in intracellular Cu concentration when comparing untreated and treated cells after 24 hours, 5.36 ± 0.13 *μ*g/g* versus *83.73 ± 2.89 (*p* = 4.61 × 10^−5^), and 48 hours 4.76 ± 0.09 *μ*g/g* versus *275.12 ± 15.62 (*p* = 5.03 × 10^−4^), respectively (Figure 2(a)). Afterwards, nuclei were isolated from both treated and untreated cells and the purification was verified by measurement of the relative amounts of the exclusively nuclear protein histone H3 and the mainly cytosolic protein SOD by Western blot (Figure 2(b)). After all these procedures, GFAAS revealed that in fact Cu entered MCF10A nuclei, based on values of Cu concentration determined in the nuclei from controls and treated cells, 2.36 ± 0.20 *μ*g/g* versus *14.55 ± 3.01 *μ*g/g (*p* = 6.03 × 10^−3^, Figure 2(c)).

### 3.2. Real-Time PCR Analyses of Cyclins D1 and B1 in Epithelial Cell Line MCF10A

**Figure 3:**
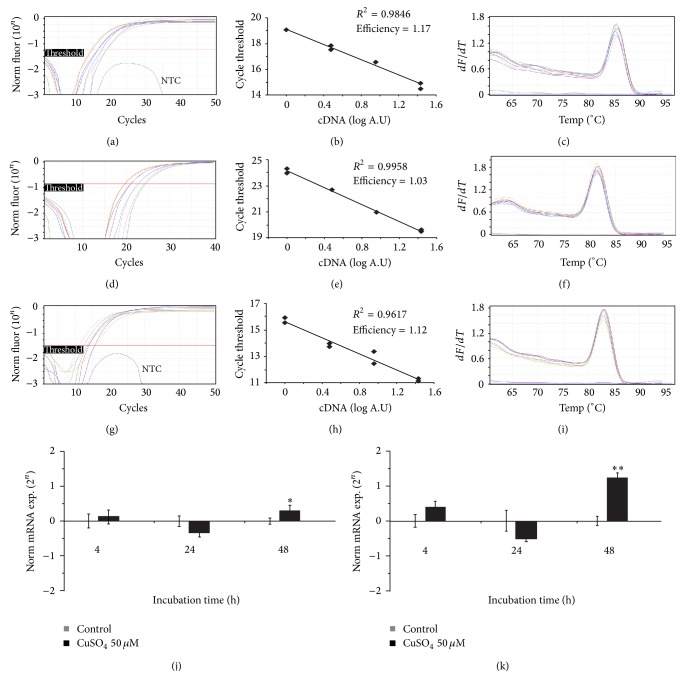
Real-time PCR revealed that Cu stimulated mild upregulation of cyclin D1 and cyclin B1 genes in MCF10A cells. Expression of cyclin genes in cells treated with 50 *μ*M CuSO_4_. The results were plotted and normalized with respect to respective untreated cells. The specificities of the primers for human glyceraldehyde 3-phosphate dehydrogenase (GAPDH), cyclin D1, and cyclin B1 were verified by real time PCR. Amplification plots, linear regression, and dissociation melting curves for (a–c) cyclin D1, (d–f) cyclin B1, and (g-h) GAPDH obtained with serial dilutions of cDNA (1, 1/3, 1/9, and 1/27). (j) Expression of cyclin D1 in Cu-treated MCF10A cells. Significant differences between untreated and Cu-treated cells are indicated by asterisks (^*∗*^ *p* < 0.05, ^*∗∗*^ *p* < 0.001).

**Figure 4:**
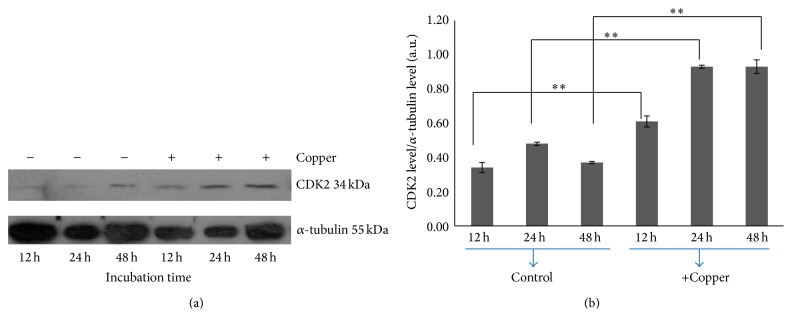
Western blot indicated that the expression levels of Cyclin-Dependent Kinase 2 (CDK2) was upregulated during Cu exposure in MCF10A cells. (a) CDK2 protein levels in cells treated with 50 *μ*M CuSO_4_ and comparison with *α*-tubulin (representative Western blots from *n* = 3). At each time point, 100 *μ*g of proteins from the total cell lysates was loaded onto each lane for the detection of CDK2. *α*-tubulin was used as loading control, and the densitometry of each lane (represented as bars) was calculated using the Image J software. (b) When compared to controls, treated cells showed augmented CDK2 levels after 12 hours. The data are expressed as arbitrary units and represent the mean ± SD of *n* = 3 independent experiments, ^*∗∗*^ *p* < 0.001.

In order to assess the mechanism by which Cu(II) salt stimulates cell proliferation in human epithelial cells, we investigated the regulation of genes involved in cell cycle progression. Specific primers were designed and their reliability verified on the basis of amplification plots obtained with serially diluted cDNA (1, 1/3, 1/9, and 1/27), linear regression analyses, and dissociation melting curves. With these procedures, we validated specific primers designed for human cyclin D1 (Figures 3(a)–3(c)) and cyclin B1 (Figures 3(d)–3(f)) as well as for GAPDH (Figures 3(g)–3(i)) as internal control. Real-time PCR revealed that exposure of MCF10A cells to 50 *μ*M of CuSO_4_ triggered upregulation of both cyclin D1 (0.297-fold induction; 23%; *p* = 1.31 × 10^−2^, Figure 3(j)) and cyclin B1 (1.236-fold induction; 136%; *p* = 2.96 × 10^−6^, Figure 3(k)) gene expression after 48 hours. Western blots obtained using mouse anticyclin dependent kinase 2 (anti-CDK2) and mouse anti-*α* tubulin (internal standard) as primary antibodies revealed that CDK2 expression levels were upregulated in MCF10A cells in the presence of Cu after 48 hr (Figure 4).

### 3.3. Analyses of ROS Generation in Normal MCF10A Cells

**Figure 5:**
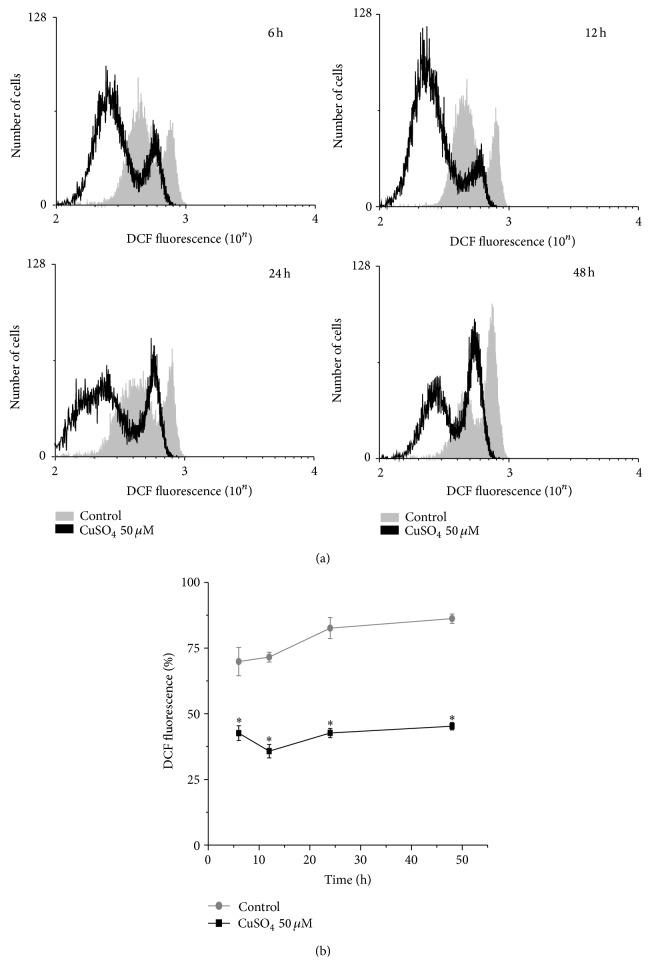
Cu-treated MCF10A cells produced less reactive oxygen species (ROS) than their untreated counterparts. The generation of intracellular ROS, cells that had been exposed to Cu(II), was estimated using the membrane-permeable nonfluorescent cell probe DCFH. (a) Graph representing typical distribution of number of cells according to DCF levels, in both controls and treated cells elicited by 50 *μ*M CuSO_4_ at specific time points, as determined by flow cytometry. (b) When compared to controls, treated cells showed reduced DCF levels after 6 hours, 12 hours, 24 hours, and 48 hours. Significant differences between untreated and Cu-treated cells are indicated by asterisk (^*∗*^ *p* < 0.01).

The generation of intracellular ROS, in the form of hydrogen peroxide and oxygen-derived hydroxyl and carbonate free radicals, by cells that had been exposed to Cu(II) was estimated using the membrane-permeable nonfluorescent cell probe DCFH, which in the oxidized form (2′,7′-dichlorofluorescein; DCF) is fluorescent [26, 35–37].  Figure 5(a) shows the changes in DCF fluorescence, measured at 520 nm by flow cytometry, during exposure of DCFH-treated MCF10 cells to 50 *μ*M CuSO_4_. As early as 6 hours, fluorescence intensity in untreated MCF10A cells was significantly higher when compared to that observed in Cu(II)-treated cells, 69.85 ± 5.40%* versus *42.62 ± 2.79% (*p* = 1.09 × 10^−4^). We also observed decrease of fluorescence intensity when comparing controls to treated cells after 12 hours, 71.57 ± 1.86%* versus *35.75 ± 2.55% (*p* = 4.81 × 10^−7^), as well as after 24 hours, 82.60 ± 4.02%* versus *42.67 ± 1.73% (*p* = 1.74 × 10^−6^). Changes in the fluorescence intensity between controls and treated cells persisted after 48 hours, 86.20 ± 1.79%* versus *45.25 ± 1.40% (*p* = 3.05 × 10^−8^), respectively (Figure 5(b)).

### 3.4. Quantification of the Intracellular Glutathione Levels

**Figure 6:**
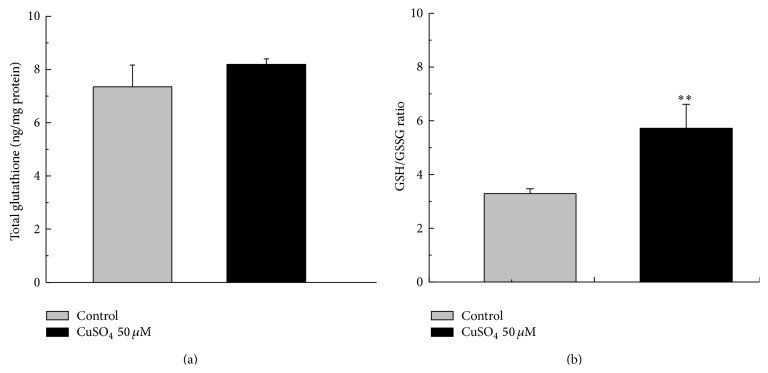
Cu treatment altered the redox potential of MCF10A cells. (a) Total glutathione in control and 50 *μ*M CuSO_4_ treated cells after 48 hours of incubation. (b) Ratio of reduced to oxidized glutathione (GSH/GSSG) after 48 hours of incubation. Significant differences between untreated and Cu-treated cells are indicated by asterisk (^*∗*^ *p* < 0.01).

In order to clarify the effects of Cu entrance in MCF10A cells, the GSH/GSSG ratios in cells that had been exposed to Cu(II) for 48 hours were determined as suggested by Estrela et al. [38]. We measured the total glutathione level in cell to ensure the level of this endogenous antioxidant was not changed during the Cu treatment. Control and Cu-treated cells exhibited glutathione total level of 7.35 ± 0.76 and 8.19 ± 0.21 (*p* = 0.1375), respectively, indicating no significant changes in the total glutathione (GSH + GSSG, Figure 6(a)). Cells of MCF10A that had been incubated on control medium for 48 hours exhibited GSH/GSSG ratio of 3.20 ± 0.05 (Figure 6(b)). Inclusion of CuSO_4_ in the medium increased to 5.72 ± 0.86 (*p* = 0.00896) the GSH/GSSG ratio.

## 4. Discussion

Cu complexes can induce apoptosis in cancer cells as a result of damage inflicted to the organelle [19, 39, 40], and this process could engender misinterpretation of the actual effect of the free metal excess on the cell cycle of normal cells. In the present study, the culture medium was supplemented with the free salt CuSO_4_ in order to investigate the influence of free intracellular Cu on the proliferation of normal epithelial MCF10A cells* in vitro*. The specific choice of the free Cu ion and its concentration was based on results recently reported by Carvalho Do Lago et al. [21]. Also, malignant cells typically possess increased levels of oxidant species that contribute naturally to the enhancement of apoptosis [41], while the lower oxidant levels of the nonmalignant MCF10A should contribute to their resistance to Cu-stimulated cell death.

Progression through the different phases of the cell cycle is regulated by specific combinations of cyclins and CDKs. Cyclins and CDKs can be involved in processes other than that of cell proliferation, as demonstrated by the reported association between cyclin expression and cell cycle reentry leading to apoptosis in neurodegenerative processes triggered by oxidative stress [42].

In the present study, normal MCF10A cells showed a mild upregulation of cyclins D1 and B1 on exposure to Cu. Interestingly, we observed that cyclin B1 was upregulated when the internal concentration of Cu exceeded 100 *μ*g/g cells, while cyclin D1 was upregulated when Cu concentrations were within the range 150−275 *μ*g/g cells. MCF10A cells permitted the entry of Cu such that the internal concentration of the metal was sufficiently elevated to cause specific upregulation of cyclins mRNA levels. Indeed, Cu was detected in the nuclei of MCF10A cells, indicating that this ion may trigger changes in gene expression, including those related to cell cycle progression. Malignant cells typically possess increased levels of oxidant species that contribute naturally to the enhancement of proliferation [41], while the lower oxidant levels of the nonmalignant MCF10A should contribute to their resistance to Cu-stimulated cell proliferation as we observed.

The concept that redox cycling controls the mammalian cell cycle through the modulation of intracellular antioxidant/oxidant species, particularly thiol-containing molecules such as GSH, has received much consideration in the literature (for a review [15]). In cancer cells, the GSH/GSSG ratio has been shown to influence the regulation of the cell cycle, mutagenic mechanisms, DNA synthesis, growth, and multidrug and radiation resistance, and GSH levels are typically higher in tumor tissue in comparison with normal tissue [38, 43]. In the present study, we observed that internalization of Cu, induced by treatment with CuSO_4_, decreased ROS levels and increased the GSH/GSSG ratio. Cu resistance has been also observed in platinum drug resistance on cancer [44]. The exposure of Cu(II) soluble in tumor mammalian cells MCF7 led to clear increase in the proliferation of the cells due to Cu uptake and disturbances of the redox status [45].

If we compare the degree of cell proliferation, the expression of genes associated with cell cycle regulation, and the levels of ROS production and related oxidative processes, in copper-treated mammary epithelial cell lines with equivalent metabolic rates, namely, MCF7 tumor cells [45] and the epithelial MCF10A nontumor cells, we can observe different behavior between cells. The observed copper-stimulated proliferation of tumor cells was not correlated with cyclin upregulation or increased cytosolic concentration of the metal, but rather with enhanced ROS generation and elevated levels of lipid peroxidation, which gave rise to alterations in the topography of the cell membranes [45]. In contrast, we observed here in this work that copper did not stimulate the proliferation of nontumorigenic epithelial MCF10A cells, although the enhanced intracellular uptake of the metal was accompanied by a moderate overexpression of cyclins D1 and B1, and an increase in the ratio of reduced to oxidized glutathione (GSH/GSSG).

Based on our findings, we suggest that normal mammary cells respond to increased levels of intracellular Cu by triggering cyclin mRNA expression and elevating the concentration of the endogenous antioxidant GSH. In turn, increased levels of antioxidant prevent abnormal ROS formation, which could cause oxidative stress and cell death. Finally, our findings point to proteins involved in cell cycle such as cyclins and CDK2 as a novel target for Cu interaction. More detailed investigation into its molecular mechanism will be important for our understanding of Cu metabolism in normal cells. We observed here that enhanced intracellular uptake and accumulation of Cu were followed by an overexpression of cyclin D1 and cyclin B1, with an increase in the ratio of reduced to oxidized glutathione (GSH/GSSG), but without ROS production. The results presented herein provide new insights into the molecular link between Cu excess and the control of cell cycle and, consequently, the mechanism by which changes in redox balance and ROS accumulation regulate cell proliferation.
